# Participation of Older Adults in Clinical Trials for New Drug Applications and Biologics License Applications From 2010 Through 2019

**DOI:** 10.1001/jamanetworkopen.2022.36149

**Published:** 2022-10-14

**Authors:** S. W. Johnny Lau, Yue Huang, Julie Hsieh, Shenggang Wang, Qi Liu, Patricia W. Slattum, Janice B. Schwartz, Shiew-Mei Huang, Robert Temple

**Affiliations:** 1 Office of Clinical Pharmacology, Office of Translational Sciences, Center for Drug Evaluation and Research, US Food and Drug Administration, Silver Spring, Maryland; 2 Now with DataRevive USA LLC, Rockville, Maryland; 3 Department of Pharmacotherapy and Outcomes Science and Virginia Center on Aging, Virginia Commonwealth University, Richmond, Virginia; 4 Now with Departments of Medicine, Bioengineering and Therapeutic Sciences, University of California, San Francisco; 5 Center for Drug Evaluation and Research, US Food and Drug Administration, Silver Spring, Maryland

## Abstract

**Importance:**

Older age may be accompanied by changes in the pharmacokinetics or pharmacodynamics or both of medications that can result in altered safety and efficacy profiles.

**Objective:**

To assess representation of older adults in clinical trials of new drug applications (NDAs) and biologics license applications (BLAs).

**Design, Setting, and Participants:**

This cross-sectional study analyzed US Food and Drug Administration (FDA) data for NDAs and BLAs approved from 2010 through 2019. Age distribution of clinical trial participants was compared with age distribution of the US population with the disease or disorder (prevalent population). Data were from adults enrolled in registration trials for depression, heart failure, insomnia, non–small cell lung cancer (NSCLC), nonvalvular atrial fibrillation (NVAF) stroke prevention, osteoporosis, and type 2 diabetes or adults sampled from US prevalent population in community-dwelling health data. Data were analyzed from November 2020 to February 2021.

**Exposures:**

Trial enrollment.

**Main Outcomes and Measures:**

Representativeness of trial populations was assessed by the participation to prevalence ratio (PPR) defined as the percentage of patients by age group among clinical trial participants to the percentage of patients by age group among US prevalent population.

**Results:**

Data from 166 clinical trials (229 558 participants) for 44 NDAs and BLAs were analyzed. The most consistent finding was the limited enrollment of the oldest age groups, namely those 75 years and above for type 2 diabetes and NSCLC, and 80 years and above for NVAF stroke prevention, insomnia, heart failure, and osteoporosis. Adults aged 60 to 74 years were enrolled in equal or greater proportion than the US prevalent population.

**Conclusions and Relevance:**

In this cross-sectional study, underrepresentation of the oldest adults existed during evaluation of new drugs and biologics, yet the older adults may represent significant proportions of the treatment population. Closing the representation gap between clinical trial enrollment and potential treatment populations is essential for safe and effective use of new drugs and biologics.

## Key Points

**Question:**

Are older adults adequately represented in clinical trials for new drug evaluation?

**Findings:**

In this cross-sectional study, adults in the oldest age groups above age 75 years or in some therapeutic areas above age 80 years in clinical trials were not enrolled in proportion to the prevalence of selected treatment indications in the US from 2010 through 2019. However, participants between ages 60 and 75 years were reasonably proportional with the prevalence of selected treatment indications.

**Meaning:**

These results suggest that a lack of adequate representation of older adults in clinical trials for new drug evaluation can present challenges for optimal use and prescribing.

## Teaser

This cross-sectional study examines the proportions of enrollment for older adults in clinical trials for new drug and biologics license applications with the US Food and Drug Administration relative to the US population.

## Introduction

The US Census Bureau projects that by 2034 the number of people who are ages 65 years and above (77 million) will outnumber children under the age of 18 years (76.5 million) in the US.^1^ By 2060, about one quarter of the US population will be 65 years and above. The US population aged 85 years and above is currently 6 million and will triple by 2060.^2^ Older age is often accompanied by increased disease burden and use of medications,^3,4^ so that older adults are the major consumers of prescription medications.^5^ Older age may also be accompanied by changes in pharmacokinetics or pharmacodynamics or both that may alter drug safety and efficacy profiles. These factors contribute to the greater likelihood of adverse drug reactions in older adults.^6,7,8,9^ Limited inclusion of older adults in clinical trials is long-standing and may result in an incomplete picture of safety or efficacy for older adults at the time of initial clinical availability.^10^ To determine recent enrollment of older adults in clinical trials for new drug applications (NDAs) and biologics license applications (BLAs), including efficacy supplements that supported approval for marketing in the US, we collected registration clinical trial enrollment data from the internal US Food and Drug Administration (FDA) database for 7 treatment indications pertinent to older adults. We then compared the age distribution of clinical trial participants by treatment indication with the age distribution of the prevalence disease population in the US.

## Methods

### Treatment Indication and Clinical Trial Population Selection

We chose treatment indications that reflect common health conditions in older adults.^11,12,13,14^ We included all NDAs and BLAs approved for the treatment of depression, heart failure, insomnia, non–small cell lung cancer (NSCLC), nonvalvular atrial fibrillation (NVAF) stroke prevention, osteoporosis, and type 2 diabetes via oral, inhalation, or injection (intravenous, infusion, subcutaneous, or intramuscular) administration routes between January 1, 2010, and December 31, 2019. The selected drugs represented about 20% of the new molecular entities (NMEs) that might be prescribed for conditions of older adults that were evaluated during this time period. We identified pivotal trials listed in section 14 of the approved drug product labeling from the Drugs@FDA website.^15^ We excluded phase 1 trials, nonrandomized trials, pediatric trials, and trials with fewer than 50 participants. We identified 166 efficacy trials of 44 NDAs and BLAs in total (eTable 1 in the Supplement). We then analyzed the participant enrollment data of all adults (over age 18 years) in these identified efficacy trials. This study was exempt from institutional review board review and informed consent requirements because data were deidentified; reporting followed the Strengthening the Reporting of Observational Studies in Epidemiology (STROBE) reporting guideline for cross-sectional studies.

### Statistical Analysis

We extracted patient-level data on age and treatment indication from FDA’s internal electronic database of clinical trials data submitted for review. We performed analyses via R programming language version 3.6.3 (R Foundation for Statistical Computing). We used 2 methods to estimate the prevalence of treatment conditions by age group in the US (Table 1). To determine the prevalence proportion of an age subgroup within the total US prevalent population, the calculated US prevalent population within the corresponding age subgroup was divided by the total US prevalent population within all age groups. For depression, heart failure, insomnia, osteoporosis, and type 2 diabetes, data were collected on the percentage of an age subgroup with the treatment indication and then multiplied by the US population within the corresponding age subgroup from the US Census data (the specific year of US Census data was chosen to match the year[s] that the prevalence data for each condition were collected) to calculate the age subgroup specific US prevalent population.^14,16,17,18^ For NSCLC and NVAF stroke prevention, the collected age subgroup prevalence data included crude numbers.^19,20^ Thus, no adjustment of the US population from the US Census data was necessary to estimate the prevalence proportion for each corresponding age group. We then determined the percentage of clinical trial participants in corresponding age group of prevalent population via dividing the number of participants in the selected age group by the total number of trial participants.

**Table 1.** Source of Prevalence Data by Treatment Indication

Treatment indication	Source	Reference
Depression	HRS; US Census	Table 20b in Federal Interagency Forum on Aging-Related Statistics,^14^ 2020
Heart Failure	NHANES; US Census	Chart 20-4 in American Heart Association,^17^ 2019
Insomnia	NHANES; US Census	Chart 12-2 in American Heart Association,^17^ 2019
Non–small cell lung cancer	SEER programs	National Cancer Institute’s SEER database^19^
Nonvalvular atrial fibrillation stroke prevention	Commercial claims database	Naccarelli et al,^20^ 2009
Osteoporosis	NHANES	Wright et al,^18^ 2014
Type 2 diabetes	NHIS; US Census	Cowie et al,^16^ 2018

Abbreviations: HRS, Health and Retirement Study; NHANES, National Health and Nutrition Examination Survey; NHIS, National Health Interview Survey; SEER, Surveillance, Epidemiology, and End Results.

We calculated the participation to prevalence ratio (PPR), a metric used to describe representation of patients by age group in a trial relative to their representation in the US prevalent population, as:



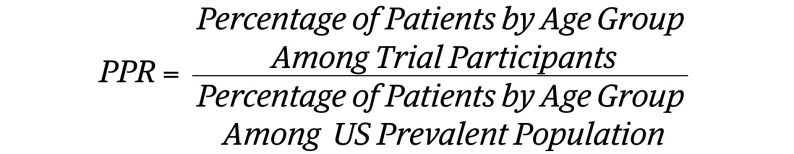



Age subgrouping in the prevalence data source dictated the age group for data comparison. We did not calculate the PPR for the depression indication because the collected prevalence data either lacked younger age subgroups^14^ or older age subgroups.^21^ Therefore, we present depression results separately.

## Results

We identified 166 clinical trials enrolling 229 558 participants that supported the approvals of 44 NDAs and BLAs for the 7 selected indications from 2010 through 2019 (Table 2; eTable 2 in the Supplement). The largest number of participants were enrolled in trials for NVAF stroke prevention (77 281 participants) and for type 2 diabetes (66 512 participants), followed by heart failure (25 918 participants), osteoporosis (25 454 participants), NSCLC (22 427 participants), depression (7977 participants), and insomnia (3989 participants). Among all trial participants, the youngest was age 18 years and the oldest was 101 years. Participants under the age of 65 years comprised approximately 50% of total trial enrollments and only 8% were above age 80 years (eTable 2 in the Supplement). The median (IQR) age within trials varied with the youngest for treatment of depression (45 [34-54] years) and the oldest in trials for prevention of stroke in patients with NVAF (72 [65-77] years). Women comprised slightly less than half of total trial participants (107 348 total participants [46.8%]) (Table 2).

**Table 2.** Summary of Clinical Trial Efficacy Population by Treatment Indications

Treatment indication	No. of NDAs or BLAs	No. of trials	Total participants, No.	Age, median (IQR) [range], y	No. (%)	
					Men	Women
Depression	4	17	7977	45 (34-54) [18-88]	2938 (36.8)	5039 (63.2)
Heart failure	2	3	25 918	64 (18-71) [18-96]	20 657 (79.7)	5261(20.3)
Insomnia	2	5	3989	58 (18-67) [18-88]	1233 (30.9)	2756 (69.1)
Non–small cell lung cancer	17	33	22 427^a^	62 (55-68) [18-93]	13 631 (60.8)	8796 (39.2)
Nonvalvular atrial fibrillation stroke prevention	4	5	77 281^b^	72 (65-77) [19-101]	48 245 (62.4)	29 035 (37.6)
Osteoporosis	4	8	25 454	70 (64-75) [20-94]	481(1.9)	24 973 (98.1)
Type 2 diabetes	11	95	66 512^c^	58 (50-64) [18-98]	35 024 (52.7)	31 488 (47.3)
Total	44	166	229 558	65 (56-73) [18-101]	122 209 (53.2)	107 348 (46.8)

Abbreviations: BLAs, biologics license applications; NDAs, new drug applications.

^a^ Included 32 missing age variables.

^b^ Included 1 missing gender variable, thus the sum of men and women is less than the total enrollment in clinical trials.

^c^ Included 19 missing age variables.

**Figure 1.**
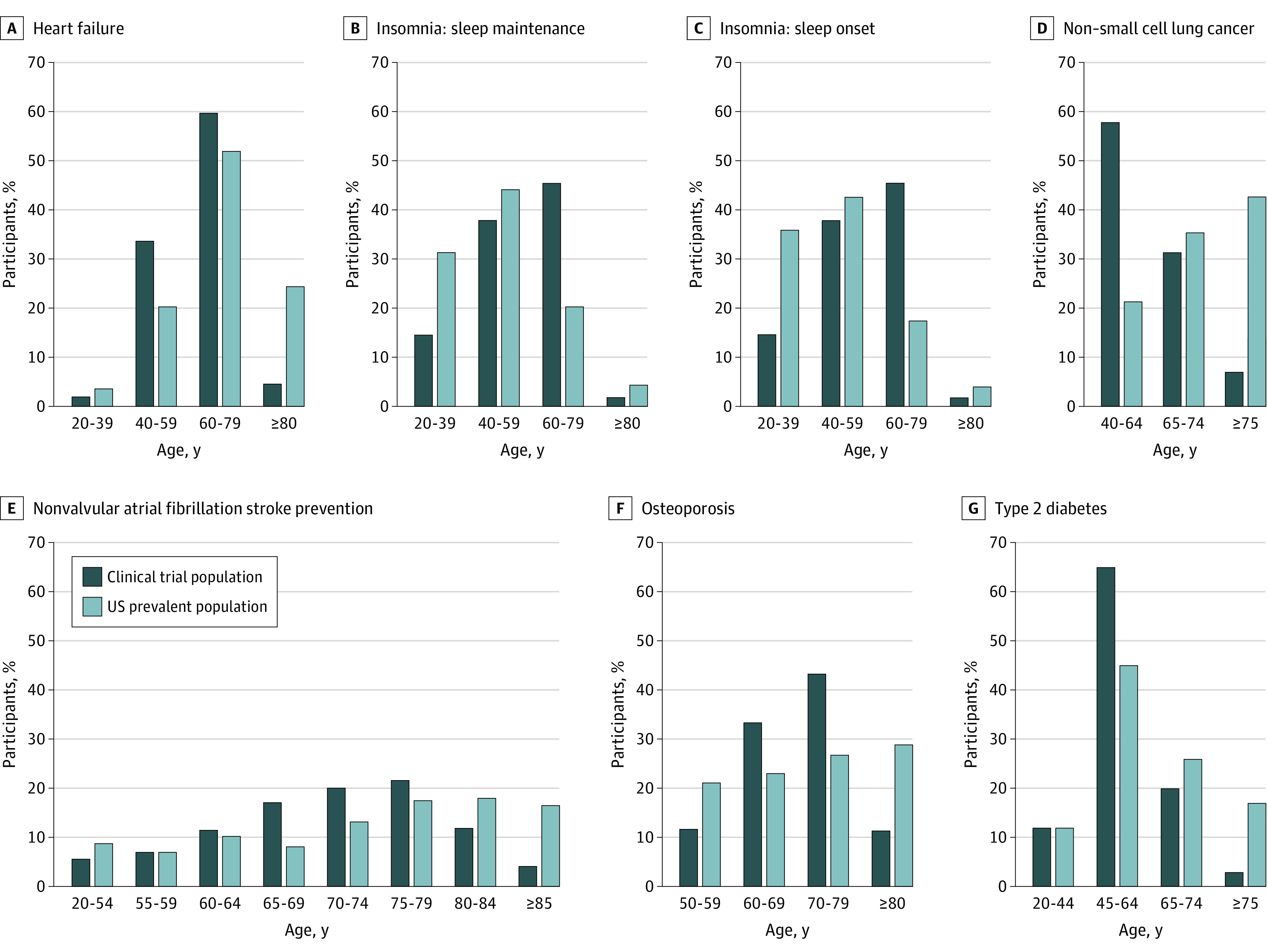
Age Distribution of Participants in Clinical Trials Compared With Prevalence of the US Prevalent Population Age subgrouping in the prevalence data source dictated the age group for data comparison.

**Figure 2.**
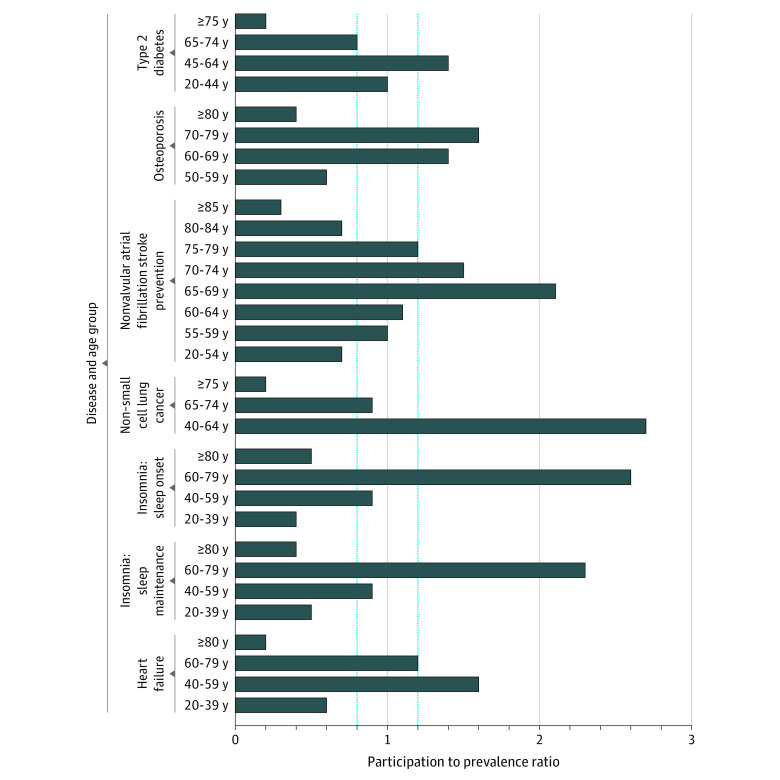
Participation to Prevalence Ratio (PPR) The blue lines indicate the PPR range of 0.8 to 1.2 often used to assess representativeness relative to the prevalent population. Age subgrouping in the prevalence data source dictated the age group for data comparison.

The age subgroups analyses showed both underenrollment and overenrollment relative to the prevalence of the treatment indication (Figure 1). For example, individuals with NSCLC who were ages 75 years and above were underrepresented (7.3% for the clinical trial data vs. 42.8% for the prevalence data), whereas individuals who were ages 40 to 64 years were overrepresented (58.0% for the clinical trial data vs. 21.5% for the prevalence data). To assess representativeness of the clinical trial enrollment, we analyzed the PPRs (Figure 2). A PPR between 0.8 and 1.2 has been suggested as a benchmark for adequate representativeness of characteristics of a trial population.^22,23,24,25^ The PPR analysis shows that participants above the age of 75 or 80 years were enrolled in trials at lower proportion than the US prevalent population. For type 2 diabetes (PPR, 0.20), heart failure (PPR, 0.19), and NSCLC (PPR, 0.17), the PPR in the above 75-year-old or 80-year-old group was 0.20 or lower. Participants in age ranges from 60 to 75 years, in contrast, were generally enrolled either in proportion to or in greater proportion than the US prevalent population. In general, participants above 80 years of age were underrepresented in clinical trials (ie, PPR below 0.5).

**Figure 3.**
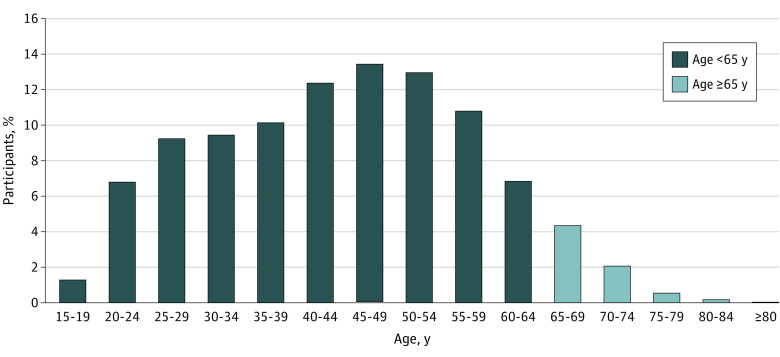
Age Distribution of Participants in Clinical Trials of Depression Numbers do not add up to 100% due to rounding.

Of 7977 clinical trial participants in trials for depression, 7417 (92.9%) were 15 to 64 years of age, and 13 (0.1%) were above age 80 years (Figure 3). Participation to prevalence comparisons across all age ranges were not feasible due to the lack of comprehensive age subgroup prevalence data. However, we can compare the clinical trial participant data with a report of US population estimates of the prevalence of clinically relevant depressive symptoms for people aged 55 and above.^14^ Among the 1960 clinical trial participants aged 55 years and above, 1400 (71.4%) were age 55 to 64 years compared with a prevalence estimate of 50%, 25% were age 65 to 74 years compared with a prevalence estimate of 27%, 2% were age 75 to 79 years compared with a prevalence estimate of 9%, and 1% were age 80 years and above compared with a prevalence estimate of 14%. The data suggest underrepresentation of older age groups in the clinical trials.

## Discussion

Our analyses focused on chronological age representativeness across the older age span in clinical efficacy trials for new drugs and biologics for 7 treatment indications during the past decade. While representation of older age subgroups varied by treatment indication, the most consistent finding was the limited enrollment of the oldest age groups. Specifically, those ages 75 years and above for type 2 diabetes and NSCLC, and 80 years and above for NVAF stroke prevention, insomnia, heart failure, and osteoporosis. The inclusion of older patients from 60 to 75 years of age in clinical trials was reasonably close to the corresponding prevalence of the treatment indication in older adults.

The FDA has developed many guidances related to drug evaluation in older adults. The 1989 Guideline for the Study of Drugs Likely to Be Used in the Elderly provides advice on the study of new drugs in older adults, encouraging routine and thorough evaluation of the drug in this specific population.^26^ The 1994 International Conference on Harmonisation (ICH; the new name is International Council for Harmonisation) Guideline E7, Studies in Support of Special Populations,^27^ also emphasized that older adult patients should be represented sufficiently to permit the comparison of drug response in them to that of younger patients. Because of the rapidly changing worldwide demographics and patterns of drug use, the ICH updated the Guideline E7 in E7 Studies in Support of Special Populations: Geriatrics Questions and Answers.^28^ The ICH E7 Guideline recommended considering older age subgroups as younger than 65, 65 to 74, 75 to 84, and over 85 years. The key points of the updated Guideline E7 included enrolling a representative number of older adult patients in clinical trials, avoiding arbitrary upper age cut-offs in trial inclusion criteria, and encouraging the inclusion of patients with concomitant illnesses.^28^ In 2020, the FDA issued guidances related to this topic, including (1) final guidance on improving the diversity of clinical trial populations to better reflect the population who will use the drug if approved, (2) a draft guidance on the adequate inclusion of older adults in cancer clinical trials, and (3) a draft guidance to assist applicants in determining the appropriate placement and content of geriatric information in labeling for human prescription drug and biological product.^29,30,31^ Our data suggest that further work is needed to ensure adequate enrollment of older adults in clinical trials and to provide the information necessary to optimize medication use in patients. Factors such as polypharmacy, multimorbidity, and organ dysfunction can contribute to the low enrollment in clinical trials for older adults.^32^ Closing the gaps in clinical trial enrollment of older adults will require engagement of multiple stakeholders, including researchers and scientific societies, regulatory bodies, health care providers, older adults and caregivers, and health care payers.^5^

Most prior studies have analyzed enrollment in clinical trials by individual patient characteristics and or individual treatment indications, and the definition for older adults has not been consistent over time.^24,26,28,31^ In 2015, the FDA established the Drug Trials Snapshots (DTS) website to show the participation of patients in trials that supported the approval of drugs and biologics by age, sex, and race. DTS also highlights whether any difference in benefits or adverse effects among these subgroups existed.^33^ DTS reports that 31% of participants in clinical trials for NMEs and biologics approved between 2015 and 2019 were at least 65 years and older.^34^ DTS currently categorizes all patients above the age of 65 as older adults and does not provide information about representativeness across the entire older age span. Similarly, most product labels provide clinical and consumer-directed information for people above the age of 65 years together. The 2020 draft FDA labeling guidance on geriatric content and placement recommends inclusion of additional information on geriatric age subgroups in drug product labeling if important differences exist in responses in older age groups.^31^ Detailed clinical trial participant age data are collected and can be reported with more granularity as could the distribution of concomitant diseases in subgroups of older adults.

Assessing age-related representativeness of a clinical trial population may still be difficult because of the limitations of available prevalence data. The prevalence data often lacked sufficient granular age data to allow comparisons across the older age span. In addition, definitions for diseases and conditions collected in surveys may not use the same standardized definitions as those for clinical trials and may not be comprehensive. National health data are collected by different federal agencies at varying intervals but are not routinely used or readily accessible for comparisons during or after drug approvals. Regular reporting, data comparability, and appropriate accessibility to the US prevalence data can help identify the age-related representativeness of clinical trial participants to fill the knowledge gaps.

Although PPR is one of the measurements to assess age-related representativeness, it has limitations. In certain situations, even if the PPR value were low, one may still be able to derive drug safety and efficacy data for particular age groups if the number of trial participants in those age groups are sufficiently large. In other situations, to improve the understanding of safety and treatment effects in some group, the specific subgroup of interest may need to be to overrepresented in the clinical trial population. Antidepressants provide such an example of a drug class that is prescribed more frequently in older adults than younger adults despite a lower reported prevalence of depression,^35^ and may have a higher rate of adverse effects in older adults due to antidepressants’ action in the central nervous system. During the evaluation of new drugs, targeted recruitment goals and even at times overrepresentation of older adults in clinical trials may be desirable to better understand the potential adverse effects of antidepressants.

### Limitations

This study had several limitations. We limited our analyses to clinical efficacy studies used to support drug approval and that appeared in section 14 of drug labels. Therefore, separate geriatric studies (such as esketamine for the treatment of depression)^36^ that did not meet primary efficacy end points or additional studies (such as cardiovascular outcome assessment of canagliflozin for the treatment of type 2 diabetes^37^ and special safety studies of lemborexant for the treatment of insomnia)^38^ were not included in our analyses. We did not analyze the data for drugs approved for antibiotics, orphan drugs, contraceptives, drugs for patients with HIV or hepatitis C virus, or for premenopausal or postpartum women. We focused on chronological age and did not address the imbalances with respect to sex^39^ or race of the study participants, which have been described independent of age by others for cardiovascular diseases including heart failure,^40^ lung cancers,^41^ osteoporosis, neurologic, and endocrine diseases,^39,42^ and other studies.^22,23,24,25^ Also, we did not compare potential imbalances in the functional status of trial participants and clinical populations. Potential imbalances in clinical trial enrollment related to sex, race, functional status, presence of multiple chronic conditions, or polypharmacy warrant further research. Finally, the clinical trial data may contain populations from non-US regions but our prevalence data for comparison was confined to published studies on the US prevalent population. Thus, the results of the comparison might not be generalizable to populations outside the US.

## Conclusions

In this cross-sectional study, the oldest age groups (above age 75 years or in some therapeutic areas above age 80 years) were not represented sufficiently in pivotal clinical trials for drug approvals in proportion to their presence in the target US treatment population. Data on safety and efficacy of medications across the entire older age span are important to appropriately care for this growing older population. Although there are long-standing FDA guidances that the clinical trial participant pool should represent the population that will ultimately receive the product,^26,29,30,31^ there continues to be room for improvement. A clinical trial ecosystem needs to be developed to acquire and provide this information so that it can be used to optimize prescribing at the time of introduction of drugs into clinical care.^5^

## Notes

Supplement. **eTable 1.** Summary List of Pivotal Clinical Trials by Treatment Indication and Generic Name **eTable 2.** Detailed Age Distribution by Treatment Indication
