# Supplementary material for: Participation of Older Adults in Clinical Trials for New Drug Applications and Biologics License Applications From 2010 Through 2019
Source: JAMA Netw Open. 2022 Oct 14;5(10):e2236149. doi: 10.1001/jamanetworkopen.2022.36149 (PMC9568796; doi:10.1001/jamanetworkopen.2022.36149)
Supplement: Supplement. — eTable 1. Summary List of Pivotal Clinical Trials by Treatment Indication and Generic Name eTable 2. Detailed Age Distribution by Treatment Indication [file jamanetwopen-e2236149-s001.pdf]

Supplemental Online Content

Lau SWJ, Huang Y, Hsieh J, et al. Participation of older adults in clinical trials for new drug applications and biologics license applications from 2010 through 2019. *JAMA Netw Open.* 2022;5(10):e2236149. doi:10.1001/jamanetworkopen.2022.36149

eTable 1. Summary List of Pivotal Clinical Trials by Treatment Indication and Generic Name

eTable 2. Detailed Age Distribution by Treatment Indication

This supplemental material has been provided by the authors to give readers additional information about their work.

© 2022 Lau SWJ et al. *JAMA Network Open.*

eTable 1. Summary list of pivotal clinical trials by treatment indication and generic name

#	Treatment Indication	Generic Name	Clinical Trial Number	Enrolled Participants, n[a]
**1**	Depression	Vilazodone	NCT00285376	397
**2**			NCT00683592	466
**3**			NCT01473394	505
**4**			NCT01473381	853
**5**		Vortioxetine	NCT00839423	313
**6**			NCT00735709	417
**7**			NCT01140906	458
**8**			NCT01153009	445
**9**			NCT01163266	457
**10**			NCT00811252	300
**11**			NCT00596817	396
**12**		Levomilnacipran[f]	NCT00969709	704
**13**			NCT01377194	557
**14**			NCT01034462	429
**15**			NCT02288325	324
**16**		Esketamine[f]	NCT02418585	236
**17**			NCT02493868	720
**18**	Heart Failure	Ivabradine	NCT02441218	6559
**19**			NCT00143507	10917
**20**		Sacubitril; Valsartan	NCT01035255	8442
**21**	Insomnia	Lemborexant	NCT02952820	949
**22**			NCT02783729	743
**23**		Suvorexant	NCT01097616	1023

© 2022 Lau SWJ et al. *JAMA Network Open.*

**24**			NCT01097629	1021
**25**			NCT00792298	253
**26**	Non-small Cell Lung Cancer (NSCLC)	Erlotinib[f]	NCT00556712	889
**27**			NCT00446225	173
**28**			NCT01328951	643
**29**		Crizotinib	NCT00932893	347
**30**			NCT01154140	343
**31**		Afatinib	NCT00949650	345
**32**			NCT01121393	364
**33**			NCT01523587	795
**34**		Ramucirumab	NCT01168973	1253
**35**		Ceritinib	NCT01828099	376
**36**		Pembrolizumab	NCT02039674	123
**37**			NCT02578680	646
**38**			NCT02142738	305
**39**			NCT02220894	1274
**40**			NCT01905657	1033
**41**			NCT02775435	559
**42**		Nivolumab	NCT01673867	582
**43**			NCT01642004	272
**44**		Gefitinib[f]	NCT00322452	1217
**45**		Osimertinib	NCT02296125	556
**46**			NCT02151981	419
**47**		Necitumumab	NCT00981058	1094
**48**		Alectinib	NCT02075840	303

© 2022 Lau SWJ et al. *JAMA Network Open.*

**49**		Atezolizumab	NCT02367781	723
**50**			NCT02366143	1202
**51**			NCT02008227	1225
**52**		Brigatinib	NCT02094573	222
**53**		Durvalumab	NCT02125461	713
**54**		Dacomitinib	NCT01774721	452
**55**		Albumin-bound paclitaxel[f]	NCT00540514	1052
**56**		Pemetrexed[f]	NCT00087711	1725
**57**			NCT00102804	663
**58**			NCT00789373	539
**59**	Non-valvular Atrial Fibrillation (NVAF) Stroke Prevention	Dabigatran	NCT00262600	18113
**60**		Rivaroxaban	NCT00403767	14264
**61**		Apixaban	NCT00412984	18201
**62**			NCT00496769	5598
**63**		Edoxaban	NCT00781391	21105
**64**	Osteoporosis	Denosumab	NCT00089791	7808
**65**			NCT00980174	242
**66**			NCT01575873	795
**67**		Bazedoxifene acetate + conjugated estrogen	NCT00675688	3101
**68**			NCT00808132	590
**69**		Abaloparatide	NCT 01343004	1645
**70**		Romosozumab-aqqg	NCT01575834	7180
**71**			NCT01631214	4093
**72**			NCT01081834	584
**73**			NCT01106677	1284

© 2022 Lau SWJ et al. *JAMA Network Open.*

**74**	Type 2 Diabetes (T2D)	Canagliflozin	NCT01809327	1186
**75**			NCT00968812	1450
**76**			NCT01032629	127[b] + 1718[c]
**77**			NCT01106625	469
**78**			NCT02025907	218
**79**			NCT01137812	755
**80**			NCT01106690	342
**81**			NCT01106651	714
**82**			NCT01064414	269
**83**		Dapagliflozin	NCT00528372	485
**84**			NCT00736879	282
**85**			NCT00859898	638
**86**			NCT00643851	598
**87**			NCT00528879	546
**88**			NCT00660907	814
**89**			NCT00680745	596
**90**			NCT01392677	218
**91**			NCT00683878	420
**92**			NCT00984867	451
**93**			NCT00673231	807
**94**			NCT02229396	694
**95**			NCT02413398	321
**96**			NCT01177813	986
**97**			NCT01159600	637[d]+ 666[e]
**98**			NCT01719003	1364

© 2022 Lau SWJ et al. *JAMA Network Open.*

**99**			NCT01422876	686
**100**		Empagliflozin	NCT01167881	1545
**101**			NCT01210001	498
**102**			NCT01011868	494
**103**			NCT01306214	563
**104**			NCT01164501	738
**105**			NCT01958671	461
**106**			NCT02033889	621
**107**			NCT01999218	1326
**108**		Ertugliflozin	NCT02099110	1233
**109**			NCT02036515	463
**110**			NCT02226003	291
**111**			NCT01986855	468
**112**			NCT00740051	227
**113**			NCT00621140	503
**114**			NCT00601250	701
**115**			NCT00798161	791
**116**		Linagliptin	NCT00622284	1560
**117**			NCT00641043	389
**118**			NCT00819091	245
**119**			NCT00602472	1058
**120**			NCT00954447	1261
**121**			NCT00800683	133
**122**			NCT00286455	329
**123**			NCT00395512	655

© 2022 Lau SWJ et al. *JAMA Network Open.*

**124**		Alogliptin	NCT01023581	784
**125**			NCT00286442	527
**126**			NCT00328627	1554
**127**			NCT00286494	493
**128**			NCT00432276	803
**129**			NCT00286468	500
**130**			NCT00286429	390
**131**		Liraglutide	NCT00294723	746
**132**			NCT00318461	1091
**133**			NCT00700817	665
**134**			NCT00856986	988
**135**			NCT00318422	1041
**136**			NCT00331851	581
**137**			NCT00518882	464
**138**			NCT00333151	530
**139**			NCT01620489	277
**140**		Lixisenatide	NCT00688701	241
**141**			NCT00763451	323
**142**			NCT01169779	391
**143**			NCT00707031	634
**144**			NCT00713830	859
**145**			NCT00763815	484
**146**			NCT00715624	496
**147**			NCT00866658	311
**148**			NCT00975286	446

© 2022 Lau SWJ et al. *JAMA Network Open.*

**149**			NCT01768559	894
**150**		Semaglutide sc	NCT02054897	388
**151**			NCT01930188	1231
**152**			NCT01885208	813
**153**			NCT02128932	1089
**154**			NCT02305381	397
**155**		Semaglutide oral[f]	NCT02906930	703
**156**			NCT02863328	822
**157**			NCT02607865	1864
**158**			NCT02863419	711
**159**			NCT02827708	324
**160**			NCT03021187	731
**161**		Insulin degludec	NCT00982644	1030
**162**			NCT01068665	457
**163**			NCT01059799	435
**164**			NCT01006291	687
**165**			NCT00972283	992
**166**			NCT01046110	447

a: Clinical trial population is collected from FDA's internal database.
b: Refers to study 1 (Add-on Combination Therapy With Sulfonylurea)
c: Refers to study 2 (Add-On Combination Therapy With Insulin (With or Without Other Antihyperglycemic Agents)
d: Refers to study 1 (Empagliflozin vs. Placebo as add-on to Metformin)
e: Refers to study 2 (Empagliflozin vs. Placebo as add-on to Metformin Plus Sulfonylurea)
f: Drugs not included as part of the FDA, CDER's annual approved new molecular entities list from 2010-2019 but approved for treatment indications during this time period.

© 2022 Lau SWJ et al. *JAMA Network Open.*

eTable 2. Detailed age distribution by treatment indication

Age Range (yrs)	No. (%) of participants enrolled in clinical trials of each indication						
	Depression (n=7977)	Heart Failure (n=25918)	Insomnia (n=3989)	Non-small Cell Lung Cancer (n=22427)	Non-valvular Atrial Fibrillation Stroke Prevention (n=77281)	Osteoporosis (n=25454)	Type 2 Diabetes (n=66512)
**15-19**	106 (1)	6 (0)	9 (0)	7 (0)	1 (0)	0 (0)	13 (0)
**20-24**	546 (7)	26 (0)	66(2)	22 (0)	4 (0)	1 (0)	106 (0)
**25-29**	735 (9)	59 (1)	131(3)	90 (0)	21 (0)	3 (0)	312 (0)
**30-34**	749 (9)	134 (1)	176 (4)	197 (1)	51 (0)	12 (0)	915 (1)
**35-39**	803 (10)	295 (1)	211(5)	407 (2)	150 (0)	13 (0)	2203 (3)
**40-44**	984 (12)	600 (2)	277 (7)	808 (4)	393 (1)	66 (0)	4309 (6)
**45-49**	1065 (13)	1193 (5)	316 (8)	1518 (7)	978 (1)	395 (2)	7394 (11)
**50-54**	1029 (13)	2180 (8)	329 (8)	2574 (11)	2922 (4)	1406 (6)	10455 (16)
**55-59**	854 (11)	4771 (18)	586 (15)	3750 (17)	5561 (7)	1552 (6)	12672 (19)
**60-64**	546 (7)	4454 (17)	451 (11)	4346 (19)	8882 (11)	3257 (13)	12397 (19)
**65-69**	342 (4)	4617 (18)	708 (18)	4130 (18)	13268 (17)	5054 (20)	8621 (13)
**70-74**	162 (2)	3828 (15)	476 (12)	2916 (13)	15540 (20)	6371 (25)	4813 (7)
**75-79**	43 (1)	2573 (10)	177 (4)	1261 (6)	16886 (22)	4465 (18)	1900 (3)
**80-84**	10 (0)	959 (4)	63 (2)	305 (1)	9309 (12)	2188 (9)	328 (0)
**≥85**	3 (0)	223 (1)	13 (0)	64 (0)	3315 (4)	671 (3)	55 (0)
**NA[a]**	-	-	-	32(0)	-	-	19(0)

The number zero "0" in parenthesis represents a rounded off number less than 0.5% of the actual value.
a: missing age

© 2022 Lau SWJ et al. *JAMA Network Open.*
